# Hydrodynamic and Sediment Responses of Open Channels to Exposed Pipe Encasements

**DOI:** 10.1371/journal.pone.0143300

**Published:** 2015-11-20

**Authors:** J. Q. Mao, H. Q. Zhang, H. C. Dai, B. H. Yuan, T. F. Hu

**Affiliations:** 1 College of Water Conservancy and Hydropower Engineering, Hohai University, Nanjing, China; 2 Zhejiang Institute of Hydraulics & Estuary, Hangzhou, China; 3 School of Earth Sciences and Engineering, Hohai University, Nanjing, China; University of Malaya, MALAYSIA

## Abstract

The effects of exposed pipe encasements on the local variation of hydrodynamic and sediment conditions in a river channel are examined. Laboratory experiments are performed to assess the response of water level, flow regime and bed deformation to several representative types of concrete encasements. The experimental conditions considered are: three types of exposed pipe encasements exposed on the bed, including trapezoidal shape, circular-arc shape and polygonal shape, and three sets of discharges, including annual discharge, once-in-3-year flood, and once-in-50-year flood. Our experiments show that: (1) the amount of backwater definitely depends on the encasement geometric shape and the background discharge; (2) smaller discharges generally tend to induce local scour of river bed downstream of the encasement, and the order of sensitivity of bed deformation to the encasement geometric shape is trapezoidal > circular-arc > polygonal; (3) comparatively speaking, the polygonal encasement may be considered as a suitable protective structure for pipelines across alluvial rivers, with relatively modest effects on the local hydrodynamic conditions and bed stabilization.

## Introduction

Subsurface pipeline crossings of open channels are widely used in gas transmission and water transfer engineering. When crossing an alluvial river, the pipeline is theoretically required to be installed below scour depth, and adequate cover must be maintained under the bed [1,2]. However, the design procedure for a pipeline crossing often does not involve a rigorous estimation of the hydrological and hydrodynamic conditions [3]. The pipes buried initially might become exposed as a result of long-term river bed degradation, which increases the risk of pipeline damage caused by currents, waves, vibration, and human activities [4–6]. The encasement method, where pipes are completely encased in a rigid jacket (e.g., reinforced concrete), has increasingly been used to protect the buried pipelines from unpredictable loads and risks. Encasement may be formed of concrete or grout in the space between the pipe and a host pipe. At river pipeline crossings, concrete encasement is the most effective and economical means of preventing pipeline failures from deterioration, corrosion or mechanical damage. In many countries like UK and Korea, when pipelines (especially gas pipelines) go through rivers or oceans, the buried pipes are required to be encased in concrete structures. The most common encasement sections used are the hollow square, rectangular and circular tubes.

If the bed exhibits drastic changes, the concrete encasement may also become partially exposed on the bed, and it actually plays a role as an unexpected obstacle beneath the surface of water. To the best of our knowledge, however, less attention has been paid to the effects of such exposed concrete encasements on the surrounding environment. This is because subsurface pipeline crossings are usually not regarded as a potential threat to the recipient rivers, since they are installed below the bed surface. However, the exposure of buried pipelines and encasements has been a commonly observed phenomenon, especially in some alluvial rivers. The Zhanghe River Pipeline Crossing, located at the Zhanghe River in Hebei Province, North China, represents a typical case of pipe encasement exposure. Its water pipelines encased in a concrete encasement were originally buried 2 m beneath the bed to convey irrigation water; however, due to river bed degradation, the concrete encasement protecting the crossing pipes has been partially exposed to the currents. This might have dual consequences: its structure may be damaged during a flood which for example has been observed in the 1996 flood event, and the exposed pipe encasement in turn changes the local flow and bed pattern, which may interfere in the natural fluvial process.

Investigations of the effects caused by different types of obstacles in channels, mainly involving piers, vegetation and hydraulic structures, have been undertaken by various researchers [7–9]. It is generally accepted that submerged or un-submerged obstacles in open channels could obstruct the flow and thus have an impact on the sediment transport and river bed evolution; the amount of influence depends highly on their geometric shape and position, as well as flow conditions of the channels. On the other hand, considerable research has been carried out to investigate the scour depth and scour pattern around pipeline systems, but limited to uncovered pipes [10–12]. Previous studies give little insight into the interaction mechanisms between exposed pipe encasements and open channel flow. Also, most of the previous studies are based on target-oriented experiments or models or approximations, implying that they cannot be directly used to demonstrate the fluvial processes related to exposed pipe encasements which are initially designed for pipeline crossing protection. For example, for the purpose of river regulation and training, a submerged dam can be constructed to raise the upstream water level, and at the same time slow down bed degradation [13]. However, the exposure of an encasement is an undesired result of river bed degradation, which might have an interference effect to the fluvial process. It is often not clear how the exposure of an encasement further changes the rivers, and what type of encasement might be appropriate for application to alluvial rivers. Therefore, understanding of the hydrodynamic and sediment responses (e.g., backwater effects, local scour, etc.) to exposed pipe encasements and optimizing the geometry of encasements are important for river training and management.

The general purpose of this study is to examine the influence of exposed pipe encasements on the local variation of hydraulic and bed conditions in a river channel. Considering that the local hydraulic conditions might be sensitive to the shapes of submerged obstacles, experimental investigations were conducted in a laboratory channel with movable bed, at various discharges with different shapes of exposed pipe encasements. The specific objectives in this study are: (1) to evaluate the sensitivity of hydraulic changes caused by exposed encasements to inflow discharges; and (2) to investigate the relation between hydraulic response and encasement shape.

The remaining sections of the paper are organized as follows. First, we study the hydraulic characteristics of upstream backwater, and the effects of upstream discharges and encasements' geometry on scour and deposition patterns downstream of the pipe encasements. Second, we discuss the possible impact of upstream flow conditions on local scour. Finally, we discuss the relation between the scour downstream and local flow energy dissipation, by analyzing the Froude number variation at the vena-contractas and local head losses over different encasements. Based on the experimental results, some suggestions for the hydraulic-optimized design of pipe encasements are given.

## Materials and Methods

### Ethics statement

No specific permits were required for the study. The study location is not privately-owned or protected in any way, and does not include a national park or other protected area of land. This study did not involve endangered or protected species of the local fauna.

### Experimental set-up and conditions

**Fig 1:**
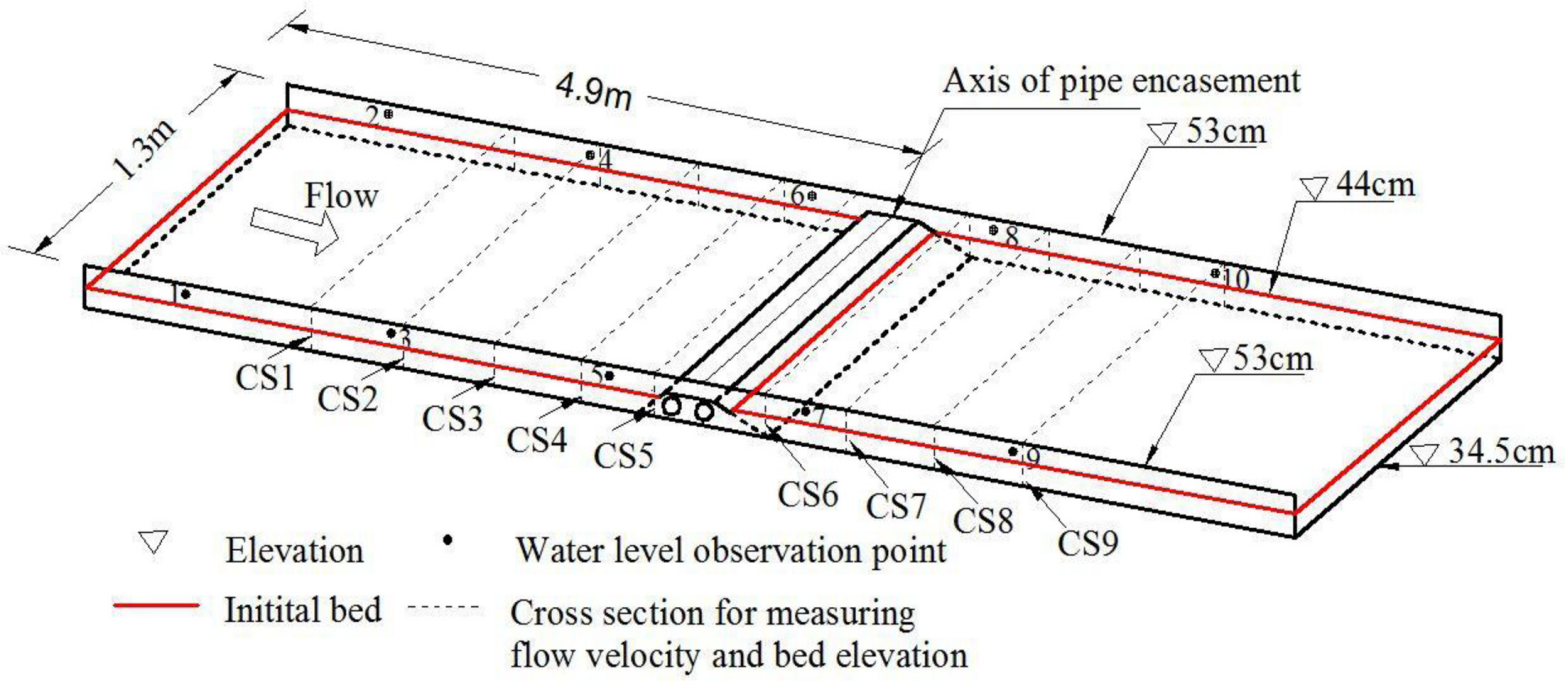
Schematic view of the model layout (not to the scale).

The experiment was designed to represent a natural channel which is interfered by an exposed pipe encasement. The designed conditions were based on direct scaling from a natural straight reach in North China, for the purposes of engineering practice. The laboratory channel was 1.3 m wide by 10 m long; the elevations of the bottom, the left and right banks were 34.5 cm, 53 cm and 54 cm, respectively (Fig 1). The channel is characterized by a flat erodible bed and fixed banks. Alluvial river beds in North China are usually made of fine sediment, and the upper sediment particles are finer than that in the lower layer [14]. The designed bottom of the channel in our experiment was covered by two sand layers, where the upper layer was 4 cm thick fine plastic sediment of *d*_50_ = 0.05 mm and the lower one was 5.5 cm thick coarse plastic sediment of *d*_50_ = 0.24 mm, respectively.

**Fig 2:**
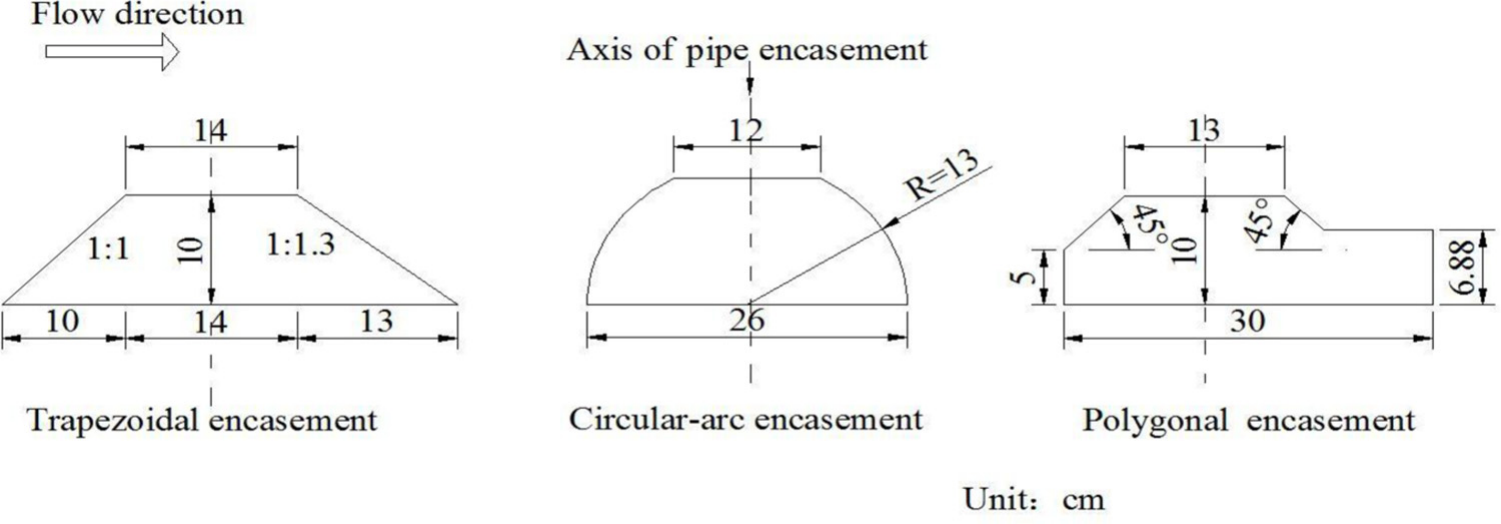
Cross-sections and dimensions of three types of pipe encasements used in the experiments.

**Fig 3:**
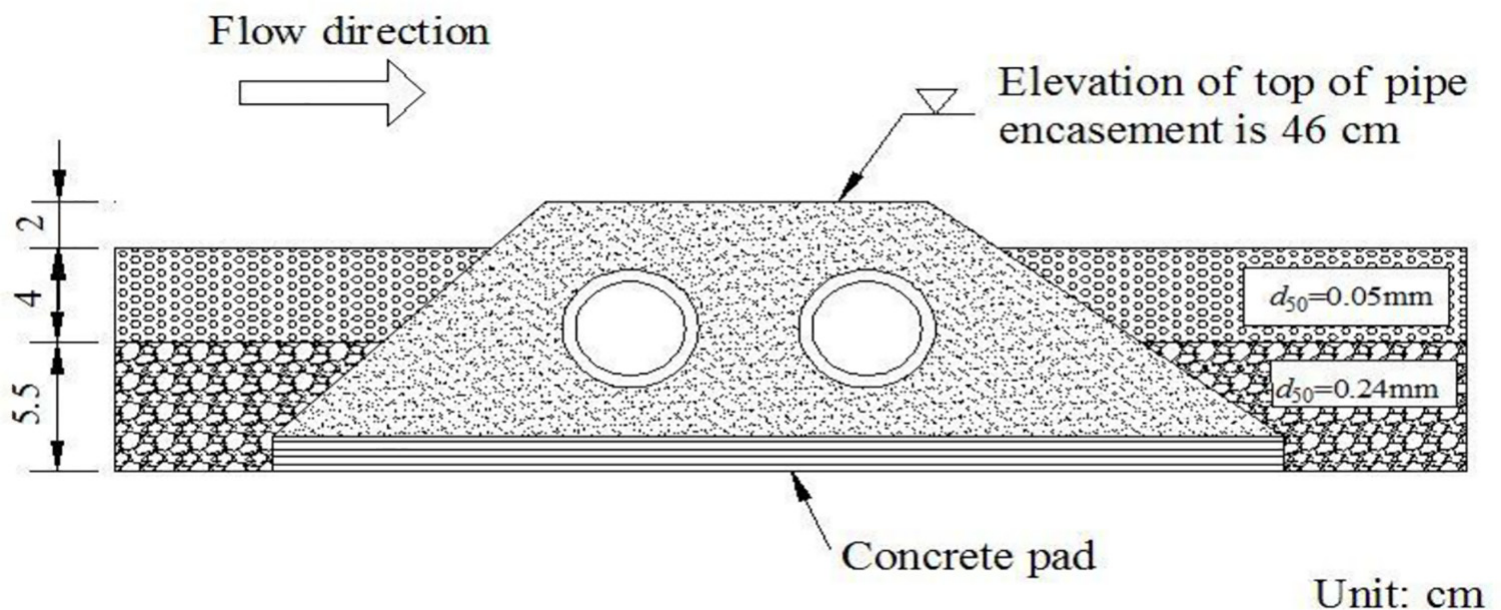
Longitudinal cross-section of the initial bed near a trapezoidal encasement.

To evaluate the hydrodynamic responses to geometric shapes of exposed pipe encasements, four different experimental phases were considered (Table 1). The difference among the phases lay in the manner of pipe encasements. In Phases I-III, three encasement configurations were respectively used: the trapezoidal shape, the circular-arc shape and the polygonal shape (Fig 2). All the pipe encasements had the same cross-sectional area which could fully encase two parallel pipes (whose diameter was 4 cm in the model). Before each test run, a specific encasement was partially buried and across the channel, 4.9 m away from the upstream boundary (Fig 1). The concrete pad under the pipe encasement was adjusted to ensure the elevation of the top of exposed encasements was 46 cm, and the distance from the encasement top to the initial bed was 2 cm. For example, Fig 3 illustrates the local configuration of the initial bed near a trapezoidal encasement. Additionally, there was no encasement (i.e., no pipeline crossing project) installed inside the channel for Phase IV, namely, it was used to provide background information on natural flow.

**Table 1:** Experimental conditions for Phases I-III (with encasements) and IV (without encasements).

Phase	Encasement	Discharge *Q* (Ls^-1^)	Sediment feed rate *V*_*s*_ (kgmin^-1^)	Experimental period *T* (min)
I	trapezoidal	2.0	0.29	55.47
I	trapezoidal	5.0	0.48	59.47
I	trapezoidal	20.7	1.91	59.47
II	circular-arc	2.0	0.29	55.47
II	circular-arc	5.0	0.48	59.47
II	circular-arc	20.7	1.91	59.47
III	polygonal	2.0	0.29	55.47
III	polygonal	5.0	0.48	59.47
III	polygonal	20.7	1.91	59.47
IV	none	2.0	0.29	55.47
IV	none	5.0	0.48	59.47
IV	none	20.7	1.91	59.47

In each experimental phase, three test cases were performed under three hydrological conditions corresponding to annual discharge (low), once-in-3-year flood (medium), and once-in-50-year flood (high), respectively. A flow meter was installed in the water supply pipe to measure the flux of water. Water flow discharging into the channel (*Q*) was controlled as 2.0, 5.0 and 20.7 Ls^-1^, respectively (Table 1). For each test run, the sediment feed consisted of coarse (*d*_50_ = 0.24 mm) and fine (*d*_50_ = 0.05 mm) sediment, which were mixed in a ratio of 3:2. The plastic sand had a specific density of 1170 kg m^-3^. Corresponding, the sediment feed rates (*V*_*s*_) were 0.29 kg min^-1^ for the low discharge condition, 0.48 kg min^-1^ for the medium discharge condition and 1.91 kg min^-1^ for the high discharge condition.

Current meters, water level gauges, and an electronic total station were used to measure flow velocity (*v*), water surface profile (*H*_*L*_), and bed elevations (*H*_*R*_), respectively. The observed cross sections were arranged both in the upstream and downstream of the pipe encasement, indexed by CS1 to CS9 (Fig 1). In the upstream reach, the distances from the pipe encasement axis to CS1-CS4 were 2.2, 1.5, 1.0 and 0.5 m, respectively, and CS5 was located 0.1 m upstream of the encasement axis. In the downstream reach, CS6 was located 0.2 m downstream of the encasement axis, and the distances from the pipe encasement axis to CS7-CS9 were 0.7, 1.2 and 1.9 m, respectively. Water level observation points (No.1-No.10) were set on the left and right banks, as shown in Fig 1.

## Results

### Backwater upstream of encasements

**Fig 4:**
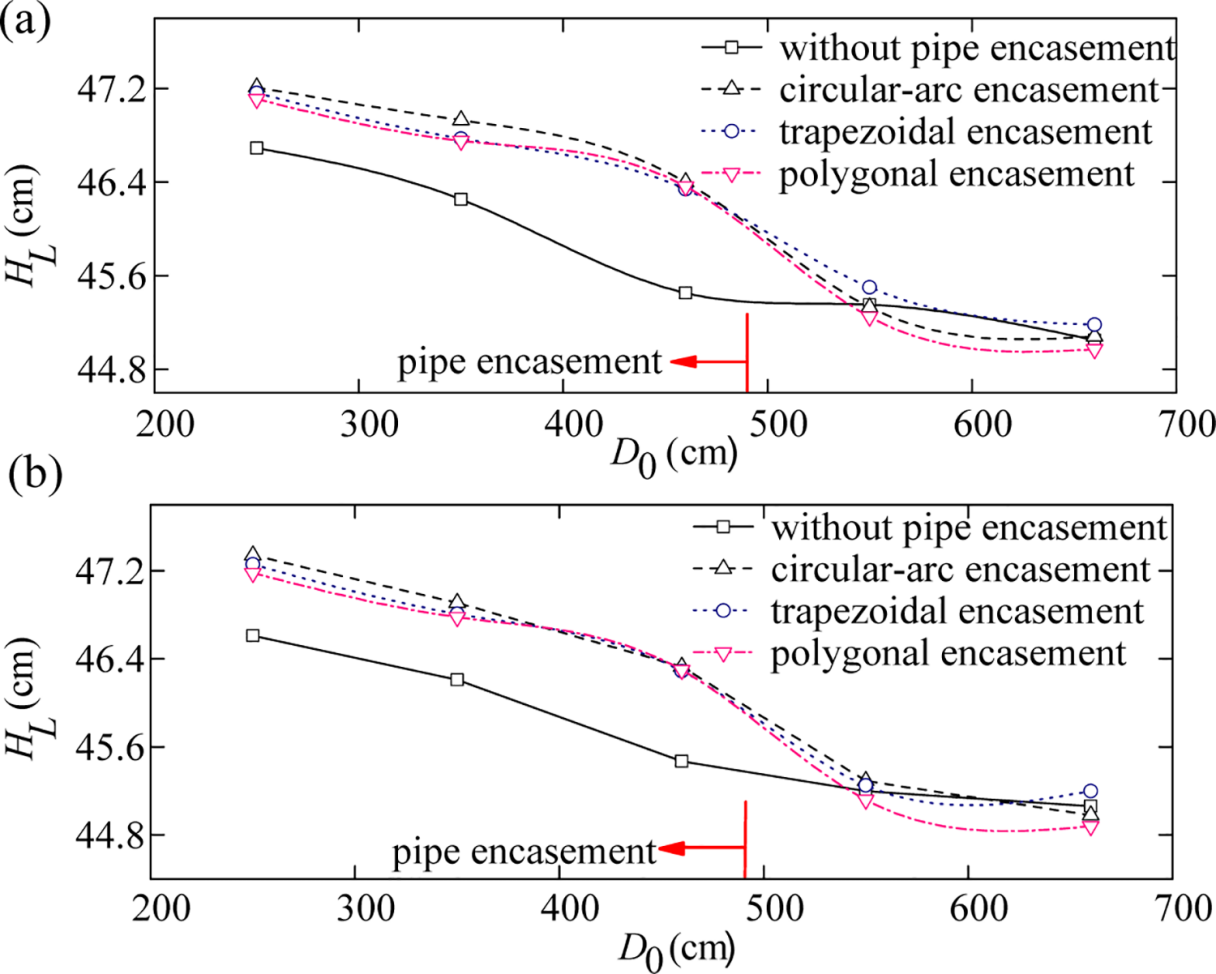
Water level curves on the left bank (a) and the right bank (b) for an annual discharge.

**Fig 5:**
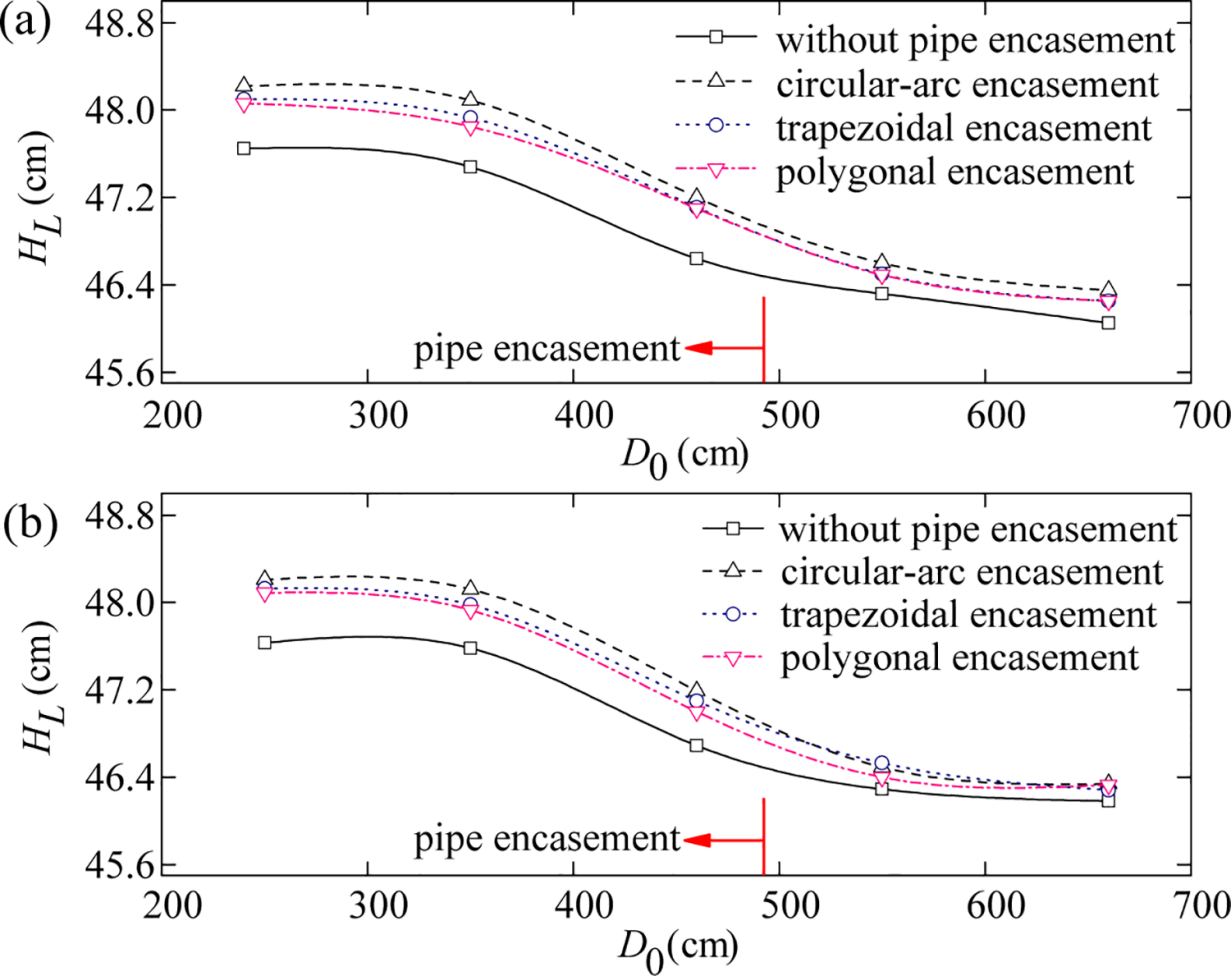
Water level curves on the left bank (a) and the right bank (b) for a 3-year flood event.

**Fig 6:**
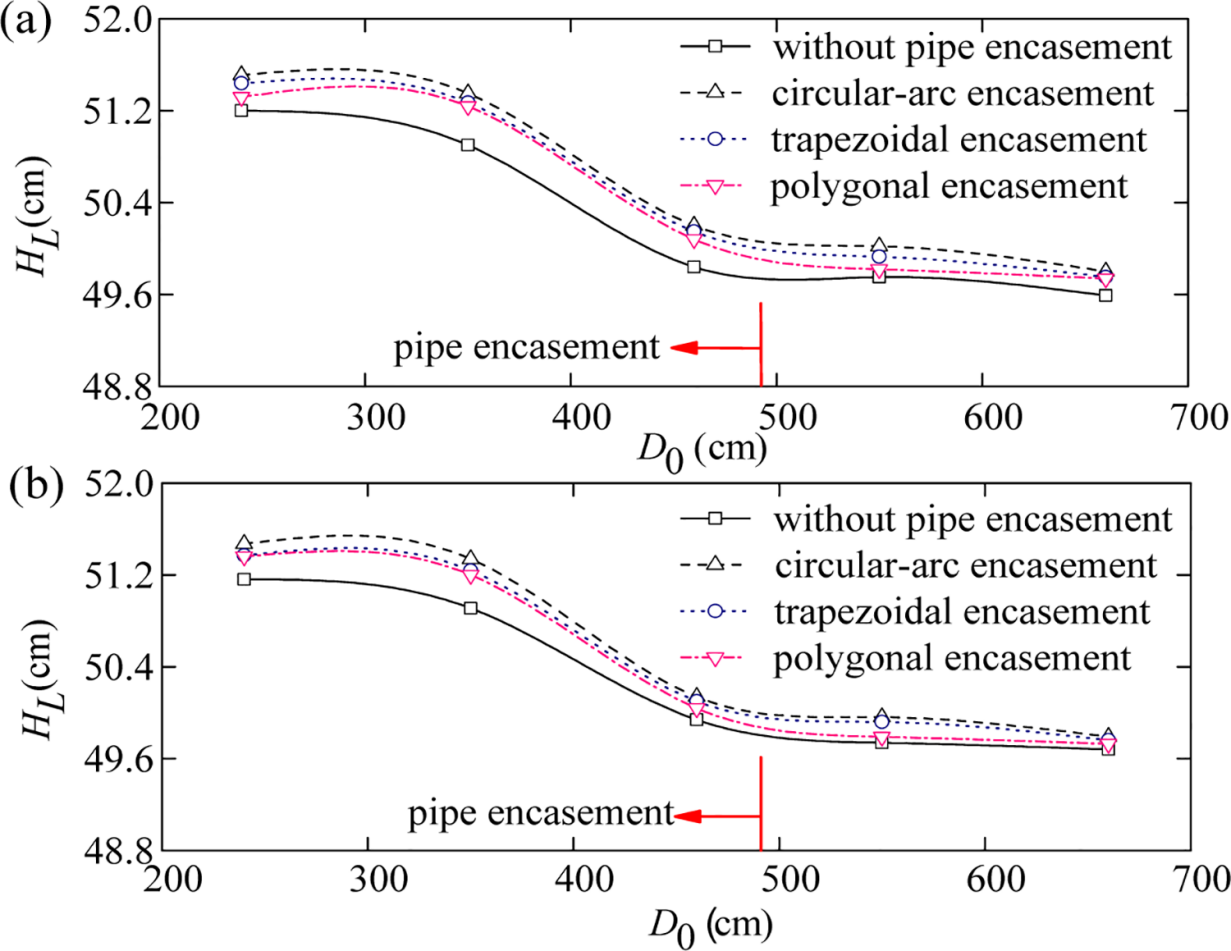
Water level curves on the left bank (a) and the right bank (b) for a 50-year flood event.

Sediment-laden water was released from the upstream boundary. For each test run, the water level profiles were measured along the left and the right banks near the pipe encasement site. Figs 4–6 show the comparison of the effects of different pipe encasements on water surface profiles under different background discharges (i.e., annual discharge, once-in-3-year flood, and once-in-50-year flood), where the abscissa (*D*_0_) is distance from the channel entrance and the ordinate (*H*_*L*_) is water level. Compared to the natural channel flow in the absence of obstruction (Phase IV), the exposed encasements (Phase I, II and III) can obstruct the flow and cause an increase in water levels upstream of the encasement, i.e. backwater phenomenon.

The amount of backwater caused by the exposed pipe encasements depends mainly on their geometric shape and inflow discharge. First, the relative amount of backwater decreases rapidly with the increase of flow discharge; for example, under the condition of annual discharge, the maximum increases in water levels caused by the circular-arc, trapezoidal and polygonal encasements are 0.855, 0.803 and 0.784 cm, respectively (Fig 4), but decreases to 0.614, 0.584 and 0.572 cm for the once-in-3-year flood event (Fig 5), and 0.390, 0.350 and 0.310 cm for the once-in-50-year flood event (Fig 6). The sites of these maximum increases in water levels are generally located at around 0.6–1.3 m upstream from the axis of pipe encasement. On the other hand, the general trend of results presented in Figs 4–6 indicates that, there is a close relation between backwater strength and encasement type. The circular-arc encasement causes a greater amount of backwater, which is possibly attributable to its relatively larger front contact area with water. The amount of backwater caused by the polygonal encasement is slightly smaller than that by the trapezoidal encasement, which is probably due to the discrepancy of their back structures.

Table 2 compares the cross-section averaged flow velocities ($\bar{v}$) of CS1-CS4 measured before and after the pipe encasements are installed. Compared to the natural flow of Phase IV, the $\bar{v}$ values decreased by around 8.4–22.0% for Phase II (with circular-arc encasements), around 7.7–17.6% for Phase I (trapezoidal), and 6.2–14.3% for Phase III (polygonal). The smaller the inflow discharge, the greater the reduction degree of the cross-section averaged flow velocity.

**Table 2:** Averaged flow velocities $\bar{v}$ of CS1-CS4 before and after the installation of concrete pipe encasements.

Flow	Cross-	No encasement	Circular-arc encasement		Trapezoidal encasement		Polygonal encasement	
discharge	section	$\bar{v}$ (ms^-1^)	$\bar{v}$ (ms^-1^)	Reduction (%)	$\bar{v}$ (ms^-1^)	Reduction(%)	$\bar{v}$ (ms^-1^)	Reduction(%)
Low ^A^	CS1	0.163	0.130	20.2	0.135	17.1	0.140	14.1
Low ^A^	CS2	0.154	0.120	22.0	0.127	17.6	0.132	14.3
Low ^A^	CS3	0.146	0.118	19.0	0.123	15.8	0.128	12.3
Low ^A^	CS4	0.135	0.114	15.6	0.117	13.3	0.120	11.1
Medium ^B^	CS1	0.288	0.240	16.6	0.249	13.5	0.254	11.5
Medium ^B^	CS2	0.273	0.224	17.9	0.233	14.7	0.240	12.1
Medium ^B^	CS3	0.252	0.216	14.3	0.216	12.7	0.224	11.1
Medium ^B^	CS4	0.240	0.208	13.3	0.210	12.5	0.214	10.8
High ^C^	CS1	0.490	0.440	10.2	0.445	9.2	0.450	8.0
High ^C^	CS2	0.475	0.432	11.9	0.440	10.2	0.442	9.8
High ^C^	CS3	0.461	0.415	9.9	0.428	9.3	0.427	7.4
High ^C^	CS4	0.450	0.412	8.4	0.415	7.7	0.422	6.2

^A^ Annual discharge

^B^ once-in-3-year flood

C once-in-50-year flood

### Bed deformation downstream of encasements

Local scour in alluvial river channels is usually found downstream of an underwater obstacle, owing to the increase in the local sediment transport capacity. The scour is a threat to the stability of gradually exposed pipe encasement mentioned above. An accurate estimate of the potential scour characteristics is thus important in the design of pipe encasements. In this study we primarily focus on the bed deformation at the cross section of CS6 immediately downstream of the crossing site, for various test conditions.

**Fig 7:**
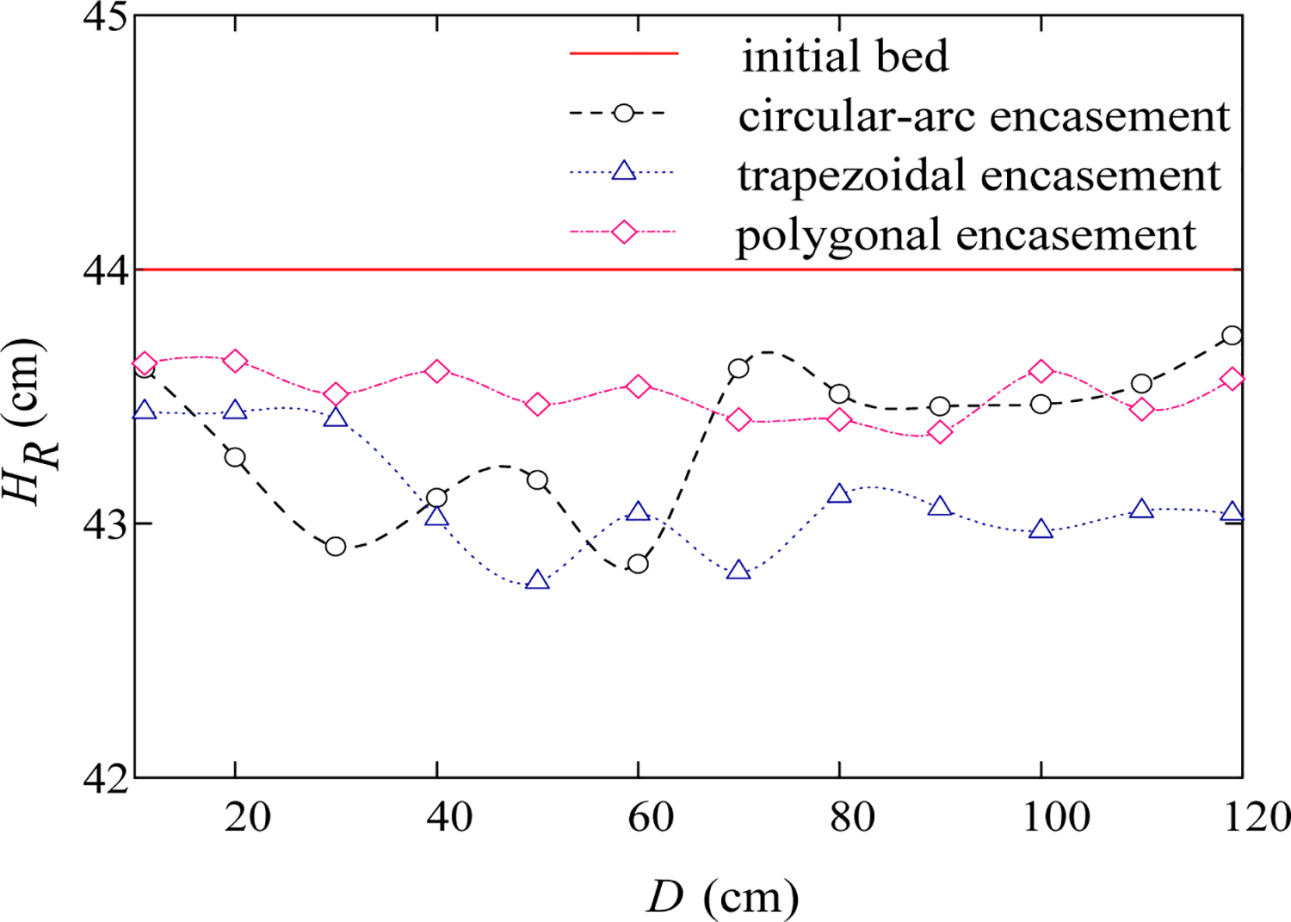
Transversal scour profile of CS6 for an annual discharge.

**Fig 8:**
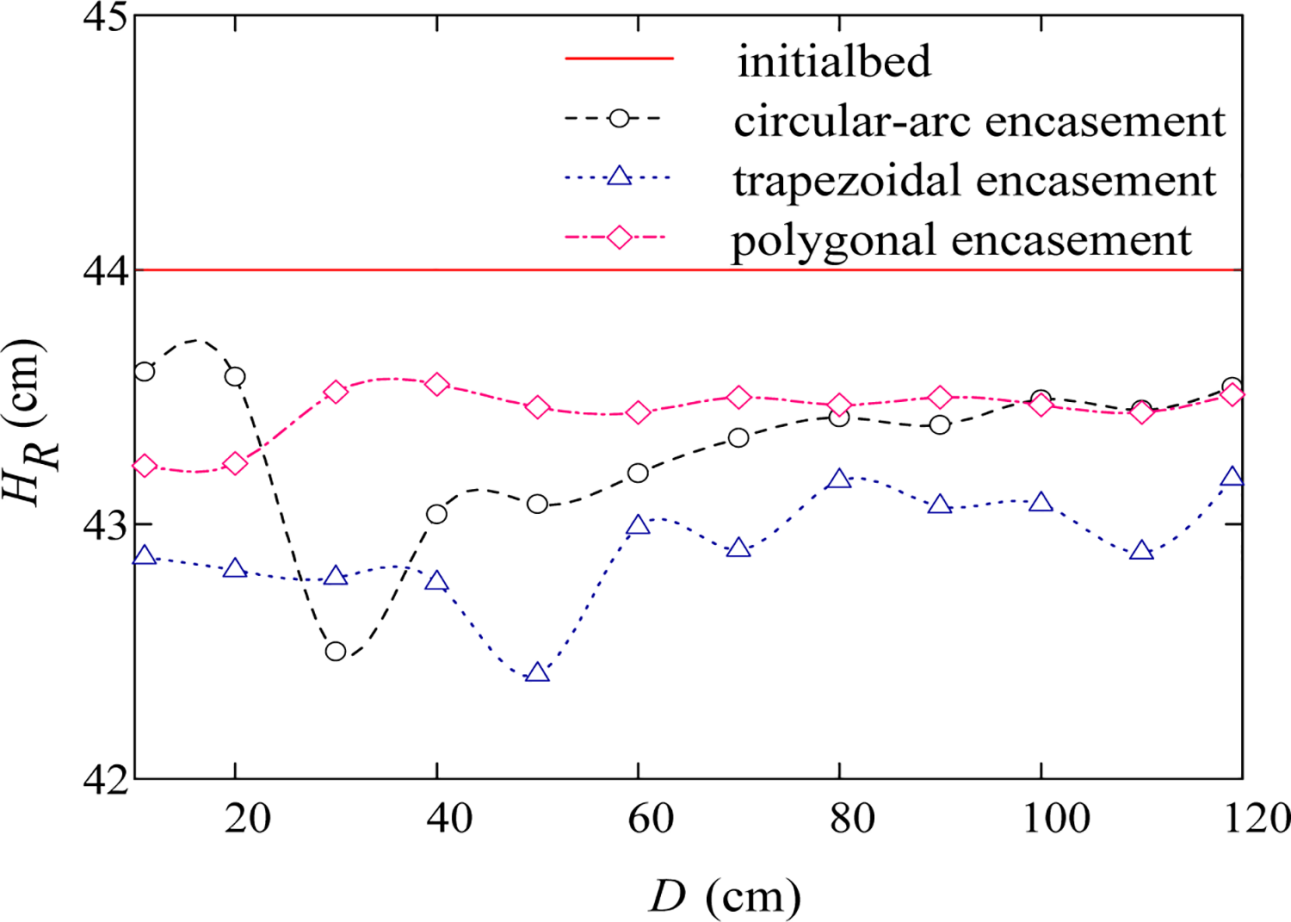
Transversal scour profile of CS6 for a 3-year flood event.

**Fig 9:**
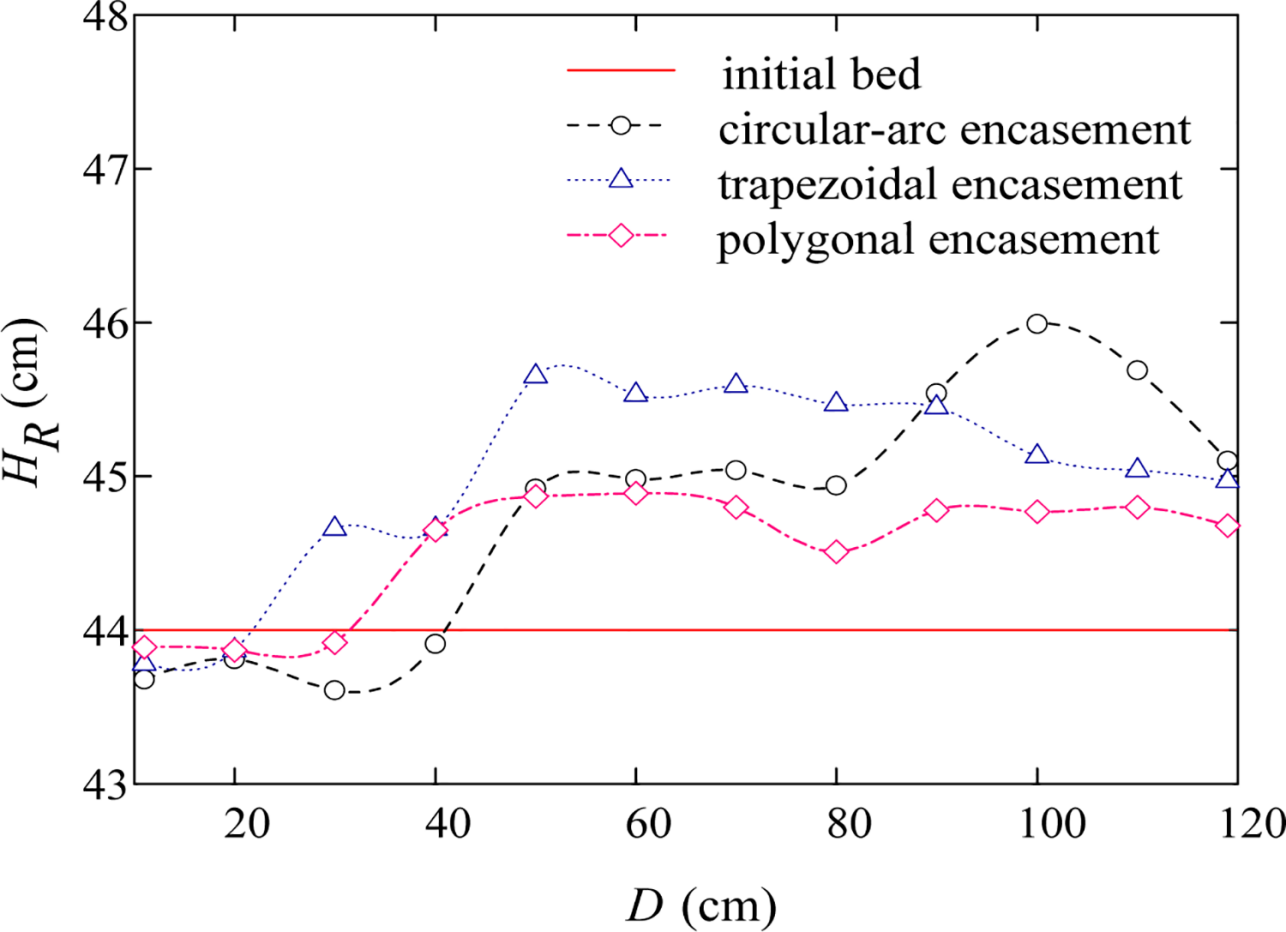
Transversal deposition profile of CS6 for a 50-year flood event.

Figs 7–9 give the transversal scour profiles of CS6 under three background dischargers, where the abscissa (*D*) is distance from the left bank and the ordinate (*H*_*R*_) is bed elevation. Comparison of Figs 7–9 reveals that, local scour is mainly observed when input flow rate is relatively small. Under the condition of annual discharge, the cross-section averaged scour depths at CS6 for the trapezoidal, circular-arc and polygonal encasement are 0.9, 0.64 and 0.48 cm, respectively; for a 3-year flood event, the corresponding scour depths can be slightly increased to about 1.1, 0.7 and 0.56 cm, respectively. As shown in Figs 7 and 8, the polygonal encasement causes an approximately flat scour cross section; for the circular-arc encasement, however, the transversal scour shape is shown to be left-right asymmetric, with relatively large scour depths (0.8–1.4 cm) near the left bank and small depths (0.5–0.6 cm) on the right side. The left-right difference in scour depth caused by the trapezoidal encasement is moderate, with the maximum scour depth of around 1.1–1.5 cm near the centre line. When the dynamic equilibrium between erosion and deposition is reached, a large quantity of sand can be transported downstream-ward due to the large background discharge (e.g., the 50-year flood event). In particular, if the incoming sediment load is more than the sediment-carrying capacity of the flow, the sediment deposition phenomenon can be observed. Fig 9 shows that the averaged deposition thicknesses at CS6 for the trapezoidal, circular-arc and polygonal encasements are 0.98, 0.77 and 0.54 cm, respectively. Our experimental results indicated that, the evolution of bed deformation profiles downstream of the exposed encasement is generally subjected to flow conditions, and the order of sensitivity of bed deformation to the encasement geometric shape is: trapezoidal > circular-arc > polygonal.

### Characteristics of flow regimes

Rivers are subject to continuous change in geometry due to unsteadiness of flow and sediment transportation through interaction between the flow and erodible boundaries [4]. Similarly, the pattern of local scour around a submerged obstacle is also mainly determined by its surrounding flow and sediment characteristics as well as the object shape. Moreover, the disturbance of exposed encasements makes the hydrodynamic behaviour more complex than normal.

**Fig 10:**
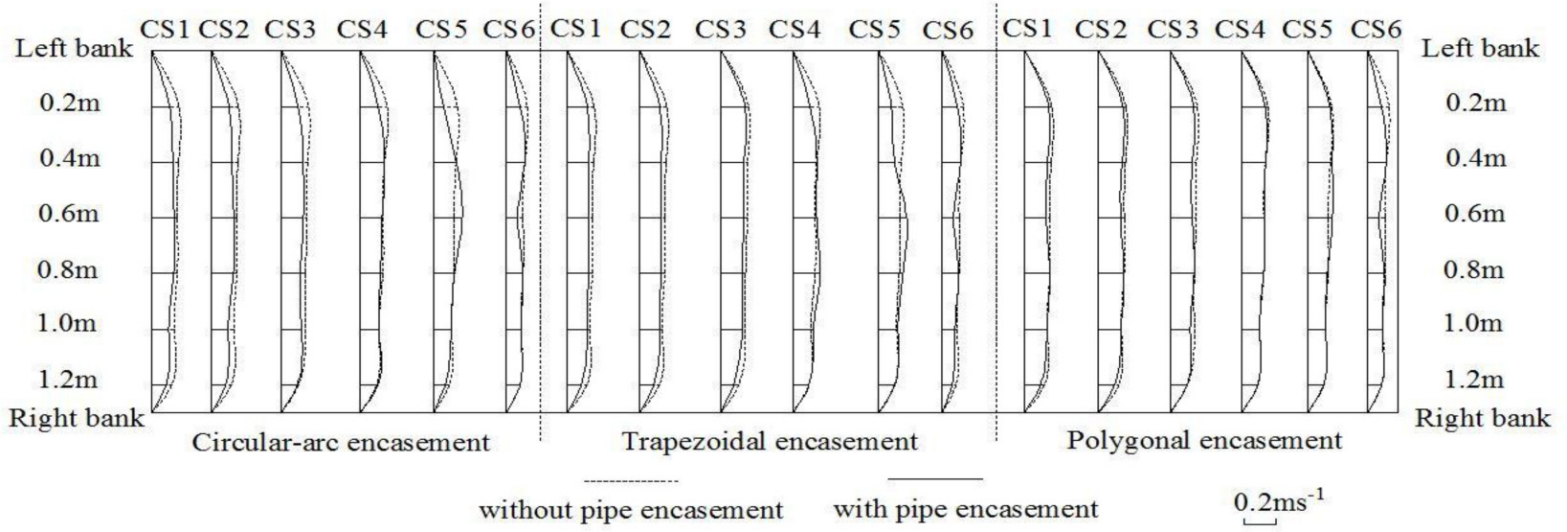
Distributions of flow speeds at CS1-CS6 for an annual discharge.

**Fig 11:**
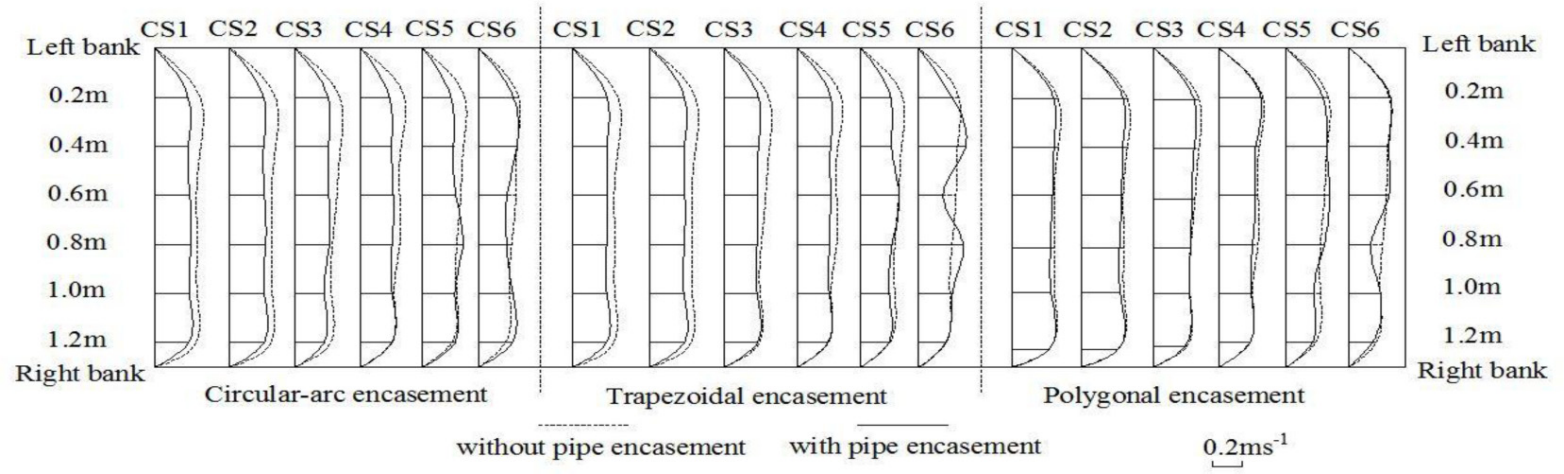
Distributions of flow speeds at CS1-CS6 for a 3-year flood event.

**Fig 12:**
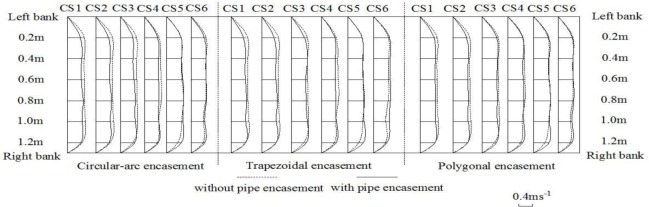
Distributions of flow speeds at CS1-CS6 for a 50-year flood event.

Figs 10–12 give the spatial distributions of flow speeds for four different experimental phases with three background dischargers. The results in the case of low discharges show that, along the flow direction from CS1 to CS5, the initially uniform flow trends to become non-uniform in the presence of exposed pipe encasement. In particular at CS5 immediately upstream of the pipe encasement, it is obvious to observe that, the flow rate in the middle channel become larger (Figs 10–11). Local bed deformation in front of the obstacle should be responsible for this similar behaviour in contraction flow; local scour can be observed at the upstream junction between the obstacle and the channel side wall, under the conditions of both annual discharge and once-in-3-year flood. In terms of the effects of pipe encasement shape on upstream flow regime, there is a marked difference in transversal speed distribution curves before and after the presence of the trapezoidal or the circular-arc shape. However, the disturbance of polygonal encasement on cross-sectional flow distribution seems to be relatively weak. We speculate that this is because the trapezoidal and the circular-arc encasements have a relatively longer front slope extending upstream-ward, leading to increased turbulence and consequent bed deformation there. For a 50-year flood event as shown in Fig 12, we observed that eroded sediments are carried from the upstream reach and trend to deposit by the encasement site, but generally the effect of exposed encasement on hydrodynamic conditions is not significant.

When water flows over the exposed encasement, the downstream flow becomes increasingly unstable. Such a phenomenon can be commonly observed under low discharge conditions. For example, both Figs 10 and 11 show the wave-like flow structures at cross section CS6, while it is not very significant for a relatively high discharge case (Fig 12), which is accordance with the bed deformation patterns (scour or deposition) presented in Figs 7–9. Meanwhile, at the same discharge, the stronger the non-uniform flow is, the larger the extent of local scour is. Among the three shapes of encasements, the trapezoidal shape produces a highly non-uniform flow at CS6, and the polygonal shape has the least effect on downstream hydrodynamic conditions. The experimental results suggest that, there is a certain relationship between flow regime and river bed deformation, and the smaller the flow discharge, the larger the impact of encasement shape on the flow regime.

## Discussion

### Analysis of water flow energy

When water flows over an obstacle, energy dissipation can be obtained due to increase in the local resistance coefficient. A hydraulic jump might occur in case supercritical flow changes to subcritical flow. Characterized by a sharp rise in the free surface elevation and strong turbulence splashing, the hydraulic jump has often been used to enhance energy dissipation in river training works. On the other hand, it is believed that local bed deformation is closely related to hydraulic jump-like flow [15,16]. In this paper, when a concrete encasement is exposed on the bed, it actually plays a role as a submerged obstacle in the open channel. For the purpose of shape optimization for crossing pipeline’s encasement design, it is necessary to analyze the characteristics of energy dissipation caused by different pipe encasements.

**Fig 13:**
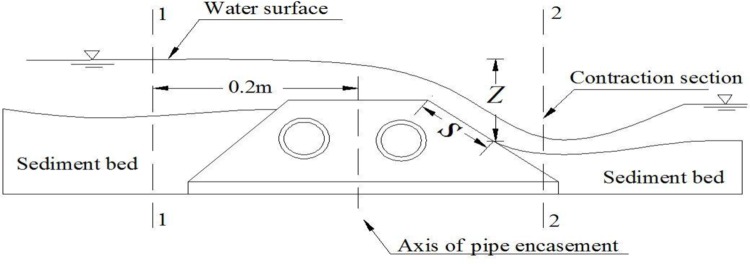
Layout of cross-sections used for calculating total water head and Froude number.

We focused on the flow energy transformation for the annual discharge and once-in-three-year flood conditions, as river bed scouring downstream of the pipe encasement mainly occurs under relatively small discharges. The Froude number (F) and total water head (*E*_0_) at control cross sections around the pipe encasement are used as the primary variables. Fig 13 shows the cross sections concerned of which Section 1–1 is 0.2 m upstream of the encasement axis and Section 2–2 is defined at the vena-contracta (i.e., the site with the lowest water level) downstream of the pipe encasement.

The water depth of the vena-contracta (Section 2–2) can be numerically estimated from Eq (1) [17]:

$$E_{0}=\Delta H_{1-2}+\frac{{\bar{v}}_{0}{}^{2}}{2g}=h_{c}+\frac{q^{2}}{2g\varphi^{2}h_{c}{}^{2}}$$ (1)

where *E*_0_ is total water head, *ΔH*_1-2_ is elevation difference between surface of Section 1–1 and bottom of Section 2–2, ${\bar{v}}_{0}$ is averaged flow velocity of Section 1–1, *g* is gravitational acceleration, *h*_*c*_ is water depth at the vena-contracta, *q* is unit discharge, and φ is velocity coefficient for a pipe encasement which could be calculated by Eq (2) (the derivation is shown in S1 Appendix):

$$\varphi^{2}=1-0.014\frac{S^{0.767}Z^{0.5}}{q}$$ (2)

where *S* is exposed length of downstream slope of a pipe encasement when flow and sediment transport stably (Fig 13) [18]; *Z* is elevation difference between surface of Section 1–1 and the level of the end of *S* (Fig 13).

Then, Froude number (F) at the vena-contracta can be given by Eq (3):

$$\text{F}=\frac{q}{\sqrt{gh_{c}{}^{3}}}$$ (3)

Table 3 compares the Froude numbers at vena-contractas (Section 2–2) for different encasements, when the background flow rate is set to the annual discharge value. The F values are 4.46 and 4.37 for the phases of circular-arc (Phase II) and trapezoidal (Phase I) encasements installed, respectively. A larger F value of 4.55 can be obtained for the phase of existence of an exposed polygonal encasement (Phase III), implying that a more stable hydraulic jump might be formed in its downstream reach. It is noted that the sequence of F values here is largely in accordance with the level of bed deformation downstream of encasements (Fig 7). Table 4 shows the same sequence of F values during a once-in-three-year flood event, but all the values are smaller than 4.5, indicating that low Froude hydraulic jump might be formed. It is usually implied that its scouring downstream should be stronger than that of annual discharge condition, which can be proved by Figs 7 and 8.

**Table 3:** Estimated parameters at the vena-contracta for an annual discharge.

Encasement	*E*_0_ (m)	*q* (m^2^s^-1^)	*S* (m)	*Z*_0_ (m)	φ	*g* (ms^-2^)	*h*_*c*_ (m)	F
Circular-arc	0.0327	0.00154	0.1081	0.0315	0.84	9.8	0.00230	4.46
Trapezoidal	0.0332	0.00154	0.1240	0.0320	0.82	9.8	0.00233	4.37
Polygonal	0.0324	0.00154	0.1040	0.0305	0.85	9.8	0.00227	4.55

**Table 4:** Estimated parameters at the vena-contracta for a once-in-three-year flood event.

Encasement	*E*_0_ (m)	*q* (m^2^s^-1^)	*S* (m)	*Z*_0_ (m)	φ	*g* (ms^-2^)	*h*_*c*_ (m)	F
Circular-arc	0.0428	0.00385	0.1218	0.0400	0.92	9.8	0.00457	3.98
Trapezoidal	0.0434	0.00385	0.1440	0.0409	0.91	9.8	0.00459	3.95
Polygonal	0.0424	0.00385	0.1200	0.0389	0.93	9.8	0.00454	4.02

Meanwhile, Bernoulli's equation for CS5 and CS6 around the pipe encasement can be written as:

$$\bar{H_{5}}+\frac{{\bar{v}}_{5}{}^{2}}{2g}=\bar{H_{6}}+\frac{{\bar{v}}_{6}{}^{2}}{2g}+\Delta \bar{H_{5-6}}$$ (4)

where $\bar{H_{5}}$ and $\bar{H_{6}}$ are averaged water levels of CS5 and CS6, respectively; ${\bar{v}}_{5}$ and ${\bar{v}}_{6}$ are averaged flow velocities of CS5 and CS6, respectively; $\Delta \bar{H_{5-6}}$ is the energy loss between CS5 and CS6, which can be assumed as $\Delta \bar{H_{5-6}}=\xi {\bar{v}}_{5}{}^{2}/2g$, where ξ is coefficient of local resistance.

The estimated results about energy loss are shown in Table 5 for an annual discharge and in Table 6 for a once-in-three-year flood event, respectively. It is shown that smaller flow discharges cause the larger degree of influence of obstacles on local head loss. Also, we estimated that the order of the impact of encasement on local head loss is: polygonal > circular-arc > trapezoidal, suggesting that the polygonal encasement is more efficient with respect to energy dissipation.

**Table 5:** Estimated parameters at CS5 to CS6 for an annual discharge.

Encasement	$\bar{H_{5}}$ (m)	${\bar{v}}_{5}$ (ms^-1^)	$\bar{H_{6}}$ (m)	${\bar{v}}_{6}$ (ms^-1^)	$\Delta \bar{H_{5-6}}$ (m)	ξ
Circular-arc	0.4627	0.1150	0.4540	0.083	0.0090	13.60
Trapezoidal	0.4622	0.1167	0.4557	0.085	0.0068	9.90
Polygonal	0.4620	0.1190	0.4526	0.084	0.0099	14.00

**Table 6:** Estimated parameters at CS5 to CS6 for a once-in-three-year flood event.

Encasement	$\bar{H_{5}}$ (m)	${\bar{v}}_{5}$ (ms^-1^)	$\bar{H_{6}}$ (m)	${\bar{v}}_{6}$ (ms^-1^)	$\Delta \bar{H_{5-6}}$ (m)	ξ
Circular-arc	0.4705	0.2180	0.4669	0.2140	0.0037	1.60
Trapezoidal	0.4700	0.2240	0.4676	0.2200	0.0025	0.90
Polygonal	0.4699	0.2270	0.4656	0.2140	0.0047	1.82

### Summary of effects of encasements

In order to minimize the hydrodynamic and sediment responses of open channels, a suitable pipe encasement should have less effect on backwater, flow regime, and bed deformation. In general, a relatively small backwater upstream of the encasement, a small scour depth downstream of the encasement, and a weak disturbance on the flow around the exposed encasement are desirable. In addition, a proper encasement shape is characterized by its capability to enhance energy dissipation (large Froude number at the vena-contracta and large local head loss), which is helpful for river bed stability downstream.

Based on the experimental results and discussions mentioned above, we obtain the order of effects of different pipe encasement shapes, with respect to upstream backwater, flow regime, bed deformation and energy dissipation. As shown in Table 7, if a polygonal encasement has to be exposed to water, both the amount of upstream backwater and its disturbance on flow regime are relatively small, and the river bed deformation near the polygonal encasement are the least. Table 7 also presents that the polygonal encasement has a stronger capability of energy dissipation, according to the comparison of Froude numbers and local head losses. Overall, the results conclude that the polygonal shape should be more suitable than the others.

**Table 7:** Summary of sensitivity of hydraulic response to different encasements.

Hydraulic response	Effects of encasement shapes	Optimum
Upstream backwater	circular-arc > trapezoidal > polygonal	polygonal
Flow regime	trapezoidal > circular-arc > polygonal	polygonal
Scour depth	trapezoidal > circular-arc > polygonal	polygonal
Froude number at vena-contracta	polygonal > circular-arc > trapezoidal	polygonal
Local head loss	polygonal > circular-arc > trapezoidal	polygonal
Total	trapezoidal > circular-arc > polygonal	polygonal

## Conclusions

Laboratory experiments are performed to assess the hydraulic response of water level, current and bed deformation to different inflow conditions as well as different types of concrete encasements. The observed results show that the hydraulic response of open channels, when a pipe encasement is exposed, is more significant in small discharges (e.g., annual discharge and once-in-3-year flood). Our results indicate a definite relation between hydraulic response and encasement shape. First, the polygonal shape has less effect on backwater and flow conditions compared with the circular-arc and trapezoidal shapes. Furthermore, as reflected by its larger Froude number values (at vena-contracta) and larger local head loss values, the polygonal type encasement also causes the smallest scour depth downstream of the encasement location. Our study provides a basis for the hydraulic-optimized design of concrete pipe encasements that would be used to protect pipeline crossings in alluvial-type rivers.

## Supporting Information

S1 Appendix Derivation of velocity coefficient. (DOC)
