# Supplementary material for: Hydrodynamic and Sediment Responses of Open Channels to Exposed Pipe Encasements
Source: PLoS One. 2015 Nov 20;10(11):e0143300. doi: 10.1371/journal.pone.0143300 (PMC4654487; doi:10.1371/journal.pone.0143300)
Supplement: S1 Appendix — (DOC) [file pone.0143300.s001.doc]

## **S1 File. Derivation of velocity coefficient**

When water flows over an encasement, according to boundary layer theory, the energy loss head (*H_L_*) at a certain cross-section in the downstream surface of the encasement can be calculated by Eq (A1) [18]:

$H_{L}=\frac{\delta_{3}U^{3}}{2gq}$ (A1)

where *U* is the potential flow velocity; *δ*_3_ is the energy thickness of a layer of fluid with velocity *U* which represents the loss of energy flux in the boundary layer; *q* is the unit discharge; *g* is the gravitational acceleration.

The boundary layer thickness *δ* can be calculated by Eq (A2), and the relation between boundary layer thickness *δ* and energy thickness *δ*_3_ can be described by Eq (A3) [18]:

$\frac{\delta}{S}=0.08\left(\frac{S}{K}\right)^{-0.233}$ (A2)

$\delta_{3}=0.22\delta$ (A3)

where, *S* is the length of downstream surface of the exposed encasement when flow and sediment transport stably; *K* is the roughness of 0.6096×10^-3^ m (0.002 ft) recommended for concrete.

If Eq (A2) is substituted into Eq (A3), we get:

$\delta_{3}=0.22\times0.08\left(\frac{S}{K}\right)^{-0.233}\times S=0.00313S^{0.767}$ (A4)

From Eq (A4) and Eq (A1), we get:

$H_{L}=\frac{0.00313S^{0.767}U^{3}}{2gq}$ (A5)

Suppose the potential flow velocity on a certain cross-section in the downstream surface of the encasement is described as:

$U=\sqrt{2gZ}$ (A6)

where *Z* is elevation difference between surface of Section 1-1 and the level of the end of *S* (Fig 13); the actual average flow velocity can be described as $V=\sqrt{2g\left(Z-H_{L}\right)}$. Thus, we may define the velocity coefficient (φ) as the ratio of actual velocity (*V*) to potential velocity (*U*):

(A7)

If Eq (A5) and Eq (A6) are substituted into Eq (A7), we can get:

(A8)
